# Client Experiences of a Telephone‐Delivered Intervention for Methamphetamine Use Disorder

**DOI:** 10.1111/dar.70108

**Published:** 2026-02-06

**Authors:** Rachel L. Petukhova, Annette Peart, Robyn Gerhard, Peta Stragalinos, Victoria Manning, Shalini Arunogiri, Dan I. Lubman, Jasmin Grigg

**Affiliations:** ^1^ Turning Point, Eastern Health Richmond Australia; ^2^ Eastern Health Clinical School Monash University Box Hill Australia

**Keywords:** methamphetamine, substance use disorder, telehealth, telephone‐delivered intervention, treatment barriers

## Abstract

**Introduction:**

Despite the significant harms associated with methamphetamine use both in Australia and globally, treatment uptake remains low. Telephone‐delivered interventions have potential to overcome many barriers to help‐seeking; however, their utility for treatment of methamphetamine use disorder is not well understood. This study explored the experiences and perceived impacts of a standalone, telephone‐delivered cognitive and behavioural intervention, Ready2Change, for individuals with methamphetamine use disorder.

**Methods:**

In‐depth semi‐structured telephone interviews were conducted and analysed using Framework Analysis.

**Results:**

Twenty‐seven participants (mean age = 42.0 years, standard deviation = 10.7, range 27–63) completed an interview. Four major themes arose: (i) Therapeutic connection experienced via telephone; (ii) Telephone format promoting comfort; (iii) Accessing treatment anywhere, anytime; and (iv) Treatment gains via telephone.

**Discussion and Conclusions:**

Evidence of therapeutic relationships was found in participant accounts of experiencing non‐judgemental support from counsellors. However, some participants reported in‐person treatment would create a stronger therapeutic relationship. Many participants described a sense of comfort the telephone format afforded them, permitting anonymity and facilitating openness. Participants reported the telephone modality to be highly accessible and convenient, particularly for those with multiple commitments. Participants reported various benefits, including greater insight regarding their methamphetamine use and reduced methamphetamine use. Contrastingly, some participants felt their use was too severe and complex to be treated via telephone. Telephone‐delivered interventions represent a potentially valuable initial step in the treatment trajectory for people with methamphetamine use disorder.

## Key Point Summary

- This study provides novel in‐depth accounts of telephone treatment for methamphetamine.
- Participants reported experiencing a therapeutic connection via telephone.
- The telephone modality of treatment was highly accessible and convenient.
- Participants described various treatment benefits, including reduced methamphetamine use.
- Telephone interventions are promising for engaging people who use methamphetamine.

## 1 Introduction

Methamphetamine use and its related burden of disease is a significant global concern [1]. In Australia, approximately 1.6 million people (7.5% of the population) have used methamphetamine in their lifetime, with 200,000 people reporting using in the past year [2]. Patterns of regular use have been increasing over time, with 23% reporting at least weekly use in 2023 compared to less than 10% in 2010 [2, 3]. Frequent methamphetamine use is associated with the often‐rapid development of tolerance and dependence, as well as a raft of physical, psychological and social harms [4, 5], including increased emergency department and psychiatric presentations [6]. These harms are seen particularly in regional areas where treatment services are lacking [7].

There are a variety of treatment options for methamphetamine use disorder [4]. Psychological interventions, including cognitive behavioural therapy and multi‐component programs (e.g., MATRIX model), are effective for reducing methamphetamine use and associated psychological distress, such as depression [8, 9]. However, there remain multiple individual (e.g., shame/stigma, low perceived treatment need) and structural (e.g., service availability, geographic location) barriers to accessing treatment [10, 11, 12].

The remote delivery of psychological interventions has become commonplace since the COVID‐19 pandemic. An increasing literature has examined the utility of telephone‐delivered interventions for alcohol and other drug use disorders [13, 14, 15]. While research on telephone‐delivered interventions for individuals with methamphetamine use disorder remains limited, a randomised controlled trial of telephone counselling following outpatient treatment [16], and a pre‐post evaluation of a telephone‐delivered intervention [14], both demonstrated reductions in methamphetamine problem severity. Further research into telephone‐delivered treatment for methamphetamine use disorder is warranted, as this approach has the potential to address treatment barriers [17, 18, 19].

Qualitative studies can provide insights into the subjective and contextual factors influencing treatment outcomes, identify barriers to treatment adherence and explore outcomes beyond clinical measures of efficacy, thereby supporting real‐world implementation [20]. Previous qualitative studies of telephone‐delivered interventions for substance use disorder have found participants perceive the telephone modality as convenient and affording a sense of anonymity in accessing drug treatment [18, 21, 22]. However, some limitations have also been noted, including reduced therapeutic relationship quality, privacy concerns and technical problems [21, 22]. Understanding people who use methamphetamine's experiences may help services in the future address common treatment barriers, such as fear of stigma or accessibility constraints. Although research on experiences of telephone‐delivered interventions for substance use disorder is expanding, little is known about client experiences of telephone‐delivered treatment for methamphetamine use. This paper presents the first known qualitative study examining the experiences and perceived impacts of a standalone, telephone‐delivered cognitive and behavioural intervention for individuals with methamphetamine use disorder, to understand the utility of treatment offered using this modality.

## 2 Methods

### 2.1 Study Design

This study utilised a qualitative design comprising semi‐structured telephone interviews. Participants were people with methamphetamine use disorder who participated in an Australia‐wide randomised controlled trial of the Ready2Change telephone‐delivered intervention [23]. Ready2Change is a manualised, multiple‐session cognitive and behavioural intervention embedded within a state‐wide 24/7 alcohol and other drug helpline [14]. Participants were contacted approximately weekly to receive four to six sessions of Ready2Change (typically 50 min in duration, delivered by the same counsellor), and were provided with workbooks to facilitate between session practice. All trial participants were provided with the parent trial's participant information sheet and provided verbal consent to participate in the parent trial, including a feedback interview. This study was approved by the Eastern Health Human Research Ethics Committee (E20/011/61428) and the Monash University Human Research Ethics Committee (25907). Reporting of this study followed the Standards for Reporting Qualitative Research [24] (Supporting Information S1).

### 2.2 Participant Recruitment and Eligibility

Participants were recruited to the parent trial from across Australia predominantly via social media (e.g., Facebook). Eligibility to participate in the trial included being 18+ years of age, experiencing mild to moderate methamphetamine use disorder (assessed using the Structured Clinical Interview for DSM‐5 Research Version, Structured Clinical Interview for the Diagnostic and Statistical Manual of Mental Disorders, Version 5—Substance Use Disorders [25]; score range 0–11 [0–1 not meeting clinical threshold; 2–3 mild; 4–5 moderate; 6+ severe], with scores 6+ warranting clinical review for inclusion), a desire to reduce methamphetamine use, and allocation to the active intervention. See protocol for detailed trial inclusion and exclusion criteria [23]. Participants were eligible to be interviewed after completing the parent trial's primary quantitative outcome assessment (i.e., 3‐month post‐randomisation), so as not to interfere with data collection. Additionally, only trial participants who completed at least one Ready2Change session were included in this analysis. Interviewers utilised convenience sampling methods to invite participants (via text message and/or telephone call) to take part in an interview. Not all trial participants were invited to participate in an interview; rather, invitations were sent when researchers had greater work capacity (e.g., less work on other projects). Interviews were scheduled via text message and/or phone call. Participants received a AUD$10 electronic supermarket voucher as reimbursement. Those who did not respond to the invitation did not provide a reason for non‐participation.

### 2.3 Measures

Participants' demographic information, methamphetamine use, and problem severity information (i.e., Drug Use Disorder Identification Test, Structured Clinical Interview for the Diagnostic and Statistical Manual of Mental Disorders, Version 5—Substance Use Disorders) [25, 26] were collected at trial baseline (i.e., pre‐intervention). All baseline data were collected and managed using REDCap electronic data capture tools hosted by Eastern Health [27].

Semi‐structured telephone interviews were conducted between August 2021 and February 2023. Interviews were recorded using a digital recorder and uploaded to a password‐protected cloud drive. The interview schedule (Supporting Information S2) included questions about experiences of the Ready2Change program and the telephone‐delivered modality, and perceived impacts of the program. Interview audio recordings were transcribed by an external transcription company and anonymised. Interviewers recorded field notes during and following the interview which described the participant's context (e.g., previous treatment and reason for seeking help), feedback on the program, perceived effectiveness of the program and interviewer reflections.

### 2.4 Data Analysis

Transcripts were analysed using framework analysis [28]. This method involves several steps: data familiarisation, indexing, charting and mapping and interpretation. Framework analysis enables the identification of themes using both inductive and deductive approaches. Data were analysed by two authors (R.L.P. and A.P.) and commenced prior to data collection ending. Data familiarisation involved reading transcripts, coding against broad themes derived from interview schedule headings, and entering key extracts into Microsoft Excel to create a draft framework. During indexing, we applied the framework to all transcript data and iteratively introduced or removed codes where necessary. The themes were clarified through the charting process, which involved manual handling of the data, discussion and agreement between R.L.P. and A.P. Finally, in the mapping and interpretation step we consolidated key codes and themes. Themes were triangulated through discussion between R.L.P. and A.P. and feedback from other members of the research team (J.G. and P.S.). Saturation [29] and information power [30] were achieved by 27 interviews. A researcher description is provided per the Standards for Reporting Qualitative Research requirements in Supporting Information S3.

## 3 Results

Twenty‐seven participants were interviewed. Interviews were completed on average 12.7 weeks (SD = 6.5) after the last completed intervention session and were a mean duration of 26.8 min (SD = 10.6). Participant demographic information is reported in Table 1.

**TABLE 1:** Participant demographic information.

Variable	Count
*N*	27
Age (mean, SD)	42.0 (10.7)
Gender	
Man	15 (55.6%)
Woman	12 (44.4%)
Geographical area	
Metropolitan	19 (70.4%)
Non‐metropolitan	8 (29.6%)
Highest level of education obtained	
Secondary	8 (29.6%)
Certificate I, II, III, IV, Diploma or Advanced diploma	14 (51.9%)
Undergraduate degree	4 (14.8%)
Postgraduate degree	1 (3.6%)
MA use problem severity (DUDIT score; mean, SD)	23.1 (5.2)
MA use disorder severity (SCID score; mean, SD)	5.8 (1.5)
Years of regular MA use (mean, SD)	13.6 (11.9)
Previous treatment for MA use	
Yes	6 (22.2%)
No	21 (77.8%)
Number of Ready2Change sessions completed (mean, SD)	4.5 (2.0)

*Note:* Mean SCID score indicates moderate substance use disorder. Two participants had SCID scores in the mild range (2–3). Ten participants had SCID scores in the moderate range (4–5). Fifteen participants had SCID scores in the severe range (6+).

Abbreviations: DUDIT, drug use disorder identification test; MA, methamphetamine; SCID, structured clinical interview for the diagnostic and statistical manual of mental disorders, version 5—substance use disorders; SD, standard deviation.

Through our framework analysis, four major themes arose: (i) Therapeutic connection experienced via telephone; (ii) Telephone format promoting comfort; (iii) Accessing treatment anywhere, anytime; and (iv) Treatment gains via telephone.

### 3.1 Therapeutic Connection Experienced via Telephone

The first theme describes participants' perspectives of the relationship built with their counsellor. Participants reported that counsellors conveyed non‐judgement and support, which contributed to the development of a therapeutic connection and, in some cases, a sense of accountability.

Overwhelmingly, participants felt their counsellors cared about them. Jane described the care conveyed by her counsellor, which distinguished the counsellor from past treatment providers:

I actually genuinely felt like somebody actually fucking cared this time […]. I'd gone through last year paying all this money for help but didn't get [support]. […] [Counsellor] called me every fucking week […] on the dot when she said she would. Every time. And I felt like she genuinely cared about it. (Woman [W], 32yo, using methamphetamine [MA] regularly for unknown years)

Robert also discussed the care he experienced from his counsellor:

[…] someone like me, sitting on my own, day in day out, left with my thoughts, feeling isolated, excluded, no connection with anyone […]. It's just great to have someone ring up that cares about me. (Man [M], 63yo, using MA regularly 13 years)

Participants also spoke of building a relationship with their counsellors, describing them as ‘like my friend’ (Kellie, F, 55yo, using MA regularly 20 years). Sandra (W, 52yo, using MA regularly 5 years) ‘questioned … how you could counsel someone on the phone with such distance’ but found it ‘*perfect*’ and was able to build rapport with her counsellor. For David (M, 48yo, using MA regularly 27 years), his counsellor's skills facilitated openness, ‘I felt comfortable talking with [counsellor] where I don't know if it was because it was over the phone, but she seemed to let me be able to open up and just express what was going on’. Jane (W, 32yo, using MA regularly unknown years) reported this connection helped her to engage with the intervention, ‘I got really attached and I liked the person who I was dealing with, which made a big difference to me too’. Mary (W, 51yo, using MA regularly 2 years) felt safe confiding in her counsellor and said: ‘[Counsellor] really, really listened and was open to everything that I was saying’. Jessica (W, 37yo, using MA regularly 22 years) echoed this sentiment and reported she perceived her counsellor as non‐judgemental, ‘[Counsellor] made me feel better about myself, she was very encouraging, she just let me be me’.

In contrast, some participants found it challenging to establish a therapeutic connection over the phone. A few compared this to in‐person treatment: ‘when you're face‐to‐face, it's more personal’ (Jessica, F, 37yo, using MA regularly 22 years). Charlie (M, 32yo, using MA regularly 11 years) also attributed a lack of therapeutic connection to the telephone intervention: ‘being face‐to‐face as well like I sort of feel that's more of a connection and probably more likely to open up and be more honest’. Some participants believed certain insights could only be gained through an in‐person interaction. The benefit of being in the same room as somebody—where ‘[a counsellor] can read you’ was missing for Jessica (W, 37yo, using MA regularly for 22 years), and also Kieran (M, 37yo, using MA regularly 14 years) stated, ‘I like to do things face‐to‐face, with anyone that I have to speak with, whether it's professionally or personally, because you get honesty, and you get the body language that you don't get when you're on the phone’.

Participants reported feeling accountable to their telephone counsellors. Paul (M, 42yo, using MA regularly 11 years) felt a sense of wanting to respect his counsellor's time and effort, ‘I didn't want to let her down … with the time she'd put in I wanted her to have an outcome’. Similarly, Grace felt motivated to continue her progress so she could tell her counsellor about her progress, ‘it was nice to have somebody there to check‐in, but also I wanted to be able to tell [counsellor], I didn't use for a whole week’ (F, 42yo, using MA regularly 8 years). Similarly, David (M, 48yo, using MA regularly 27 years) reported ‘verbal agreements with [counsellor] to do what [he] said’ helped him to stay on track.

### 3.2 Telephone Format Promoting Comfort

The second theme describes how participants perceived the telephone modality facilitated a sense of comfort in engaging with treatment. This comfort was described in terms of physical comfort, related to accessing support from their home, and the psychological comfort, related to completing sessions by telephone. For example, Jane said: ‘The phone thing worked because it was comfortable, [I] was in [my] home … It sort of suited me better than sitting down with someone and doing it. It was more comfortable for me [over the phone]’. (W, 32yo, using MA regularly unknown years).

Some participants reported feeling more comfortable participating in treatment over the telephone because it offered a degree of anonymity. Sandra discussed this in the context of living in a regional setting. Sandra had worked managing medical centres in her area previously, and ‘didn't want to go [to a local service] with the people [she] knew’. She added, *I felt comfortable because I didn't have to face someone. […], it's just a small town, … you don't have to … sit across a desk from someone that could possibly know you*. (W, 52yo, using MA regularly 5 years).

This sense of comfort extended to participant reports of being able to share more about their experiences. Anna (W, 27yo, using MA regularly 2 years) said:

I sort of have a bit of a thing where I want to put my best face [on], and almost pretend that things are fine […] whereas when it's over the phone, it's a little bit more impersonal so I feel a bit better sharing more if that makes sense.

Mary, who had never accessed help for her methamphetamine use before, explained how the telephone modality helped to address mental health factors associated with seeking support:

*I get a bit of social phobia and I have panic attacks. Sometimes I just prefer to be in my own comfortable area… I probably would have clammed up a little bit more if it was in person. So yeah, I open up more on the phone*. (W, 51yo, using MA regularly 2 years)

For Grace (W, 27yo, using MA regularly 8 years), a reason for not accessing help previously was, ‘because there's a lot of judgement and stigma’ towards people that use methamphetamine. However, Grace reported she ‘didn't feel like [she] was being judged’ during the intervention. Kellie (W, 55yo, using MA regularly 20 years) attributed this lack of perceived judgement to the telephone modality, ‘maybe I would have thought that [counsellor] would have judged me if [counsellor] would have seen me in person’.

### 3.3 Accessing Treatment Anywhere, Anytime

The third theme outlines how participants described the telephone modality as facilitating engagement in treatment. Participants reported convenience, particularly around their schedules; fewer obstacles to participating; and they could create unique therapeutic environments (e.g., private physical space) in preparation for a telephone‐delivered session.

Participants reported the telephone modality was convenient when it came to fitting sessions around the rest of their commitments. Despite initial preferences for in‐person care, Paul noted:

… at the time, I would've [chosen] face‐to‐face, but looking back on it, it was much more convenient to have it on the phone and I probably would've had to cancel a lot of sessions from not being able to make it and things happening at the last minute [if face‐to‐face]. (M, 42yo, regular MA use for 11 years)

Participants shared how they were able to integrate the intervention into their current schedules. Max (M, 33yo, regular MA use for 7 years), who was working from home at the time of the intervention, described that ‘it was very easy to integrate … It was able to work around what else I had on. It was much easier to navigate than having to try to find time to travel somewhere to meet with someone’. As part of the intervention, treatment sessions were available outside of traditional addiction treatment service hours (i.e., after‐hours and weekends), which some participants reported to value. Lachlan (M, 32yo, regular MA use for 16 years), who was working and in sole custody of his child, reported he was typically ‘running around or doing something’ during the day, and preferred to be able to ‘schedule calls like after six [pm], which was good’.

Eliminating the need for travelling to treatment came with other benefits, which was particularly prominent among participants living in regional areas. For example, Sarah (W, 27yo, regular MA use for 6 years) reported she had lost her driver's licence, and that the telephone modality of this program meant she could speak to a counsellor without ‘[having] to risk getting in the car or having to taxi’. Anna (W, 27yo, regular MA use for 2 years) described how lack of transport access means telephone treatment is more cost‐effective, ‘[face‐to‐face] can be quite expensive particularly because I don't have a car, so going out and you know, trying to find help [in‐person], you know, it's quite prohibitive’.

Participants described creating a private, therapeutic environment for themselves in preparation for appointments. Tom (M, 36yo, regular MA use for 8 years) explained how he prepared, ‘I was getting a call on the Sunday. I'd have time to go and find somewhere quiet and relaxing. I was just going over [to the cemetery], finding one of the trees and sitting under that’. There were several other accounts of curating a private space in preparation for sessions. Mary (W, 51yo, regular MA use for 2 years), who was living in her own home, described her space and the steps that she took to be prepared for her session, ‘[I was doing the call in] the lounge room because it's quiet in here … Made sure my phone is all charged and have a drink and I've got my cigarettes next to me’.

However, there are potential challenges in offering treatment via telephone in terms of clients needing to find appropriate physical space to engage with treatment (as opposed to face‐to‐face services where this space is provided). Some participants lived with other people and described how they found ways to work around this challenge. Linda (W, 51yo, regular MA use for 21 years), lived with her brother and his daughter and reported ‘I was able to arrange the calls around times when my niece wasn't home, so she didn't overhear’. Grace (W, 27yo, regular MA use for 8 years) was able to create privacy for herself despite her shared living arrangement, ‘I had a housemate so I would have to leave the house and maybe sit in my car and talk to [counsellor]’. Finally, for Carl (M, 57yo, regular MA use for unknown years), who was staying with his mother while he cared for her, he reported ‘there was need for discretion so … I'd be either lying on [my] bed or walking up and down outside in the sun’.

While many participants were able to create a private space and ensured they were free from distraction during their sessions, ‘I'm just lying on the bed, I paid full attention to [counsellor]’ (Kellie, 55‐year‐old female, using MA regularly for 20 years), some participants reported taking appointments in unconventional environments. For example, Christopher (M, 34yo, regular MA use for 7 years) shared, ‘there was one time when I was wandering around a shopping centre […] I kind of got lost. If anything, my mind was more in the call than it was in my surroundings’. Other participants described the temptation to multi‐task during a session, potentially leading to divided attention and reduced engagement. Kieran (M, 37yo, regular MA use for 14 years) described sitting at his work desk while participating in a telephone session, ‘If an important email came in, I might read through that, but my focus was generally on the call’.

Despite the accessibility of telephone‐delivered treatment, some barriers to participation remained. Christopher explained how the complexity of managing a professional job while maintaining his methamphetamine use left him without the time to benefit from the intervention:

My work takes the majority of my everything every day. The fact that I'm using the rest of the time to scurry around privately and procure large amounts of meth, it didn't leave a lot of room for me to commit. (M, 34yo, regular MA use for 7 years)

Max (M, 33yo, regular MA use for 7 years) echoed Christopher's sentiments about struggling to juggle fulltime work with engaging in treatment for his methamphetamine use. Max found he had limited capacity to engage with intervention content outside of sessions, ‘I don't have a huge amount of time in the day to do follow‐up, looking at the exercises and working through the workbook, so I probably could have got a bit more out of it in that sense’.

### 3.4 Treatment Gains via Telephone

The fourth theme relates to the outcomes that participants attributed to the intervention. This includes greater insight into when and how they use methamphetamine, changes in the frequency of methamphetamine use, and future treatment.

Some participants reported they had greater insight regarding the triggers underpinning their methamphetamine use, and the intervention ‘helped because…I started to realise where some of the urges to go and get shit were coming from’ (Tom, 36, M, using MA regularly 8 years). Mary described how the skills she learned through the intervention helped her:

A lot of things [counsellor] said really made sense, to do with my use and slowing down and triggers […]. Things that I've never even thought of and I really got a lot out of it … I wouldn't have known how to deal with the triggers … I just would have grabbed for the closest things to me which would have been the drugs. (W, 51yo, using MA regularly 2years).

Similarly, Max described acquiring ‘tools to help [reduce his use]’*:*

[The program has] certainly made me more consciously aware of what I could be doing to reduce it. I guess also a bit more aware of where it was coming from, what was causing me to use and what I could potentially be doing to reduce that. (M, 33yo, using MA regularly 7 years)

Some participants did report a reduction in their methamphetamine use, which they attributed to the intervention. Tom (M, 36yo, using MA regularly 8 years) reported he was ‘nearly six months clean’ since participating, and Jane (W, 32yo, using MA regularly unknown years) reported ‘I'm four months clean now and it actually was a really fucking good experience for me […] it literally changed my fucking life’. Anna (W, 27yo, using MA regularly 2 years) stated she was ‘currently eight weeks [abstinent], apart from one lapse’. Other participants reported while they ‘haven't stopped completely […] I've certainly benefited from the [intervention] to bring it under control’ (Carl, M, 57yo, using MA regularly unknown years). Robert, recently estranged from his family, described his progress and reduction in the frequency of his methamphetamine use since participating in the intervention:

I'm doing better, but I'm still not perfect. I still have moments when I've been ignored [by family] and sitting on my own at night, fighting with my thoughts … I'm still sometimes weak and go get some [meth]. But it's gone from every day to probably only one day or two days a week. (M, 63yo, using MA regularly 13 years)

Meanwhile, Jessica (W, 37yo, using MA regularly 22 years) shared while she was able to abstain from methamphetamine during the intervention, she lapsed after a tragic event. Jessica said, ‘one month I didn't have anything, then my mum died and that's what triggered me off. So, I was doing the right thing, I was stopping, […] I really was, and then things just triggered me mentally’. Similarly, Peter (M, 31yo, using MA regularly 5 years) shared that although he had reduced his methamphetamine use, these changes did not last longer‐term, ‘at the time I benefited and now that's behind us and I don't feel any different’.

Christopher, who like many participants in this study reported a long history of use, indicated the intervention was not able to address his addiction severity.

My level of addiction is very advanced. I'm not functioning without it … I'm going through something like three or more grams in a week and injecting it, and if I can't procure it for any reason, then I will struggle to even attend work. So, I'm very highly dependent on it, and the road back for me, I feel is somewhat long and arduous … (M, 34yo, using MA regularly for 7 years)

Indeed, some participants reported the intervention could have been more intensive, ‘at least twice a week’ (Jessica, F, 37yo, using MA regularly 22 years), and a preference for a longer duration, reporting they ‘could have got more out of it if there were a few more sessions… something more ongoing to support those changes with some longevity’ (Max, M, 33yo, using MA regularly 7 years).

Participants were able to draw on other forms of support to bolster the impact of this brief telephone delivered intervention. Tom (M, 36yo, using MA regularly 8 years) began attending mutual aid meetings ‘at about the same time’ as starting the intervention and reported it helped him gain confidence to actively engage in meetings. Tom said, ‘doing the [counselling] calls … helped me to be able to speak out in [Narcotics Anonymous] meetings’. Participating in the intervention generated a positive experience of help‐seeking for Christopher who said he was ‘probably a lot more likely now to try and get help’. Christopher continued:

Would I enlist the help of some of the organisations that are out there for this rather than going on my own? Definitely. [Counsellor] reminded me of how important counsellors and psychologists are, […] touching base once a week, and having that outlet there is so valuable. (M, 34yo, using MA regularly 7 years)

## 4 Discussion

This study sought to explore the experiences and perceived impacts of a standalone, telephone‐delivered cognitive and behavioural intervention for individuals with methamphetamine use disorder. Our analysis revealed four themes: (i) Therapeutic connection experienced via telephone; (ii) Telephone format promoting comfort; (iii) Accessing treatment anywhere, anytime; and (iv) Treatment gains via telephone.

Participants described a strong therapeutic connection with their counsellor despite the telephone modality. This connection was reflected in their accounts of receiving support and genuine care, where they felt they could share their challenges without judgement. Participants reported feeling positively towards their counsellors, working with them to meet agreed goals and building a sense of accountability. A strong therapeutic relationship is a key element of any psychological intervention and one of the strongest predictors of positive outcomes [31, 32, 33]. Our findings provide evidence that participants accessing a telephone intervention were also able to experience a strong therapeutic relationship. This is consistent with research that has found sufficient therapeutic connections can be built in telephone‐delivered interventions for alcohol use problems and other mental health concerns [18, 34, 35]. However, a small number of participants felt that face‐to‐face modalities were superior to telephone modalities in their ability to build a strong therapeutic connection. This finding is also echoed in previous research and highlights the need to account for clients' personal preferences when treatment planning [22].

The telephone modality afforded participants a sense of comfort. For many, the telephone format provided a sense of anonymity and perceived protection from stigma which can facilitate disclosure [36], similar to previous studies of telephone‐delivered interventions for alcohol use problems [18]. People who use methamphetamine experience heightened barriers to care due to perceived stigma [10, 11, 12]. However, participants in our study, who typically had high substance use severity but had not previously accessed support, reported feeling comfortable discussing their methamphetamine use with their counsellor. This suggests that telephone‐delivered interventions may appeal to underserved groups, who experience high rates of stigma and significant barriers to treatment.

Participants reported the telephone modality as easy to participate in, that they could connect to an appointment from anywhere, at any time. Practical barriers, such as being too busy or forgetting appointments, are common factors impeding engagement in treatment [12, 37]. Participants reported they were able to access the intervention with less associated ‘opportunity costs’ (such as sacrificing time away from paid work), which reduced their likelihood of missing appointments. The accessibility of telephone‐based interventions also means that people living in regional and remote areas, as some of our participants were, can receive treatment more readily.

Our findings support recent research that describes how telephone‐based interventions can accommodate the ‘life complexities’ of some people seeking addiction treatment and facilitate participation by difficult‐to‐reach cohorts [22]. Participants reported that one benefit of the intervention they received was that they were able to engage in support while being in less conventional times and locations (e.g., after‐hours, a cemetery, a car, while working from home), though this could bring distractions in the environment that could compromise engagement with treatment. A key clinical consideration in delivering interventions via this modality is supporting clients to create a quiet, private space for themselves so that they can actively participate in sessions without distraction.

Participants reported beneficial treatment outcomes, including a greater awareness of their triggers to use methamphetamine and strategies to manage cravings. Some participants reported reducing their methamphetamine use in response to the intervention, and several stopped using methamphetamine altogether. This is consistent with previous studies of stand‐alone telephone‐based interventions for alcohol or drug use [13, 14, 15], and our findings have clinical implications for telephone‐delivered treatment for substance use in general. However, some participants reported that the intervention duration was too brief to lead to noticeable and sustained change.

While telephone‐delivered interventions can be beneficial for some, the unique challenges associated with moderate to severe methamphetamine use disorder (e.g., prolonged withdrawal, cognitive deficits, high relapse risk, co‐occurring mental health issues) can necessitate more intensive, multi‐modal approaches [4]. Still, other low intensity interventions for methamphetamine use, such as telephone‐based aftercare support, text‐based interventions, and mobile applications have been associated with modest reductions in methamphetamine use [16, 38, 39]. A recent review concluded that reductions in use appeared to be more prominent among individuals who had not previously accessed treatment or who were in the early stages of seeking support [40]. We suggest that while telephone‐delivered interventions will not be sufficient for all individuals, they are highly accessible and scalable and may play an important role in methamphetamine use treatment offerings.

Participants reported that the intervention provided a positive treatment experience, which led some to accessing further support and others to report that they were more likely to do so in the future. These findings correspond to research on other low‐intensity interventions, including brief computer‐based interventions, where participants also reported accessing further support [39]. Indeed, the Ready2Change program for methamphetamine use could be an important stepping stone to future, more intensive treatment for those needing further support.

### 4.1 Strengths and Limitations

This study had several strengths, including: (i) a large qualitative sample recruited from across Australia (including many regional areas) to achieve theme saturation; (ii) the use of in‐depth semi‐structured interviews to enable rich data collection and capture clients' nuanced perspectives; and (iii) ensuring rigour by using dual coding and achieving consensus via group discussion. There were two primary limitations to this study. Firstly, participants who had a positive experience of the intervention may have been more motivated to participate in a qualitative interview. However, completing qualitative interviews via telephone enables participants to share openly even when discussing a sensitive topic, and may have helped participants feel more comfortable sharing negative feedback [36]. Secondly, qualitative interviews were completed following the primary outcome assessment (at least 6 weeks following intervention), and after three research calls had already been completed; therefore, participants may have experienced issues with recall after this period. Additionally, participants' accounts of receiving the intervention calls may have been influenced by their experiences of the non‐counselling calls with researchers who were not their counsellor.

## 5 Conclusion

The Ready2Change telephone‐delivered intervention enabled participants to experience a therapeutic connection with their counsellor and access support in ways that were comfortable and convenient. Participants highlighted many positive intervention impacts such as greater awareness of their methamphetamine use, learning strategies to manage urges, and reduced use among some participants. While the telephone modality suited most individuals in this study, some reported a preference for more intensive, face‐to‐face options. This speaks to the importance of providing a range of treatment offerings to support people with barriers to accessing in‐person care while also catering to those who need face‐to‐face support to adequately engage in treatment and achieve lasting benefits. Future work is needed to investigate how people engaging in brief interventions for methamphetamine use can be effectively supported to ‘step‐up’ treatment intensity as needed. The results of this study suggest that highly scalable programs such as Ready2Change can serve as a potentially valuable initial step in the treatment trajectory for people who experience methamphetamine use disorders.

## Author Contributions

Conceptualization: Jasmin Grigg. Methodology: Jasmin Grigg, Rachel L. Petukhova, Robyn Gerhard, Peta Stragalinos. Formal analysis: Rachel L. Petukhova, Annette Peart. Investigation: Rachel Petukhova, Robyn Gerhard. Writing – original draft preparation: Rachel L. Petukhova, Jasmin Grigg, Annette Peart. Writing – review and editing: Rachel L. Petukhova, Jasmin Grigg, Annette Peart, Robyn Gerhard, Peta Stragalinos, Victoria Manning, Shalini Arunogiri, Dan I. Lubman. Funding acquisition: Dan I. Lubman, Victoria Manning, Shalini Arunogiri, Jasmin Grigg. Supervision: Jasmin Grigg, Victoria Manning, Shalini Arunogiri, Dan I. Lubman.

## Funding

This work was supported by the National Health and Medical Research Council (1186268).

## Conflicts of Interest

This work was supported by a Clinical Trials and Cohort Studies Grant from the National Health and Medical Research Council (1186268). D.I.L., V.M., S.A. and J.G. have received grants from the National Health and Medical Research Council. D.I.L. and V.M. have received grants from the Medical Research Future Fund. D.I.L. and J.G. have received funding from Shades of Pink. D.I.L., V.M. and J.G. have received funding from VicHealth, the Victorian Department of Health, the Eastern Health Foundation, and the National Centre for Clinical Research on Emerging Drugs. D.I.L. and V.M. have received grants from the HCF Research Foundation, the Alcohol and Drug Research Innovation Agenda, the Alcohol and Drug Foundation, and the Victorian Responsible Gambling Foundation. D.I.L. has received grants from Google and the Australian Department of Health and Aged Care. D.I.L. has received funding from the Australian Research Council. V.M. has received funding from the Transport Accident Commission (Victoria). J.G. has received funding from the Victorian Department of Transport and Planning. D.I.L. is supported by a National Health and Medical Research Council Leadership Fellowship.

## Supporting information


**Supporting Information: S1:** Standards for reporting qualitative research checklist (SRQR) (O’Brien et al. 2014).

**Supporting Information: S2:** Experiences of telephone‐delivered intervention for methamphetamine use interview schedule.

**Supporting Information: S3:** Researcher description.

## Data Availability

Research data are not shared.
