# Supplementary material for: Client Experiences of a Telephone‐Delivered Intervention for Methamphetamine Use Disorder
Source: Drug Alcohol Rev. 2026 Feb 6;45(2):e70108. doi: 10.1111/dar.70108 (PMC12880202; doi:10.1111/dar.70108)
Supplement: Supplementary file 2 — Supporting Information: S2: Experiences of telephone‐delivered intervention for methamphetamine use interview schedule. [file DAR-45-0-s001.docx]

**Supplementary Material 2:**

**Experiences of Telephone-Delivered Intervention for Methamphetamine Use Interview Schedule**

*Question prompts provided in italics*

**Setting the scene**

1. Can you tell me a little bit about yourself and what prompted you to participate in the Ready2Change study?
   - *Wanted to reduce or stop meth use? Why now?*
   - *Helping research? Why is that important to you?*
   - *For reimbursement*
   - *Saw it on Facebook / other advertising*
2. Have you ever received any treatment for your drug use before the Ready2Change study?
   - If so, how long ago, which type of treatment, which drug and for how long?
   - Was this treatment in-person or via telehealth?
   - What factors influenced you to seek out this treatment at the time?
   - What was your experience of this treatment?
3. What, if anything, has stopped or made it difficult for you to seek support for your ice use?
   - *E.g. cost, location / couldn’t get there, knowledge of where to go, no time, long wait, embarrassed, thought it would get better by itself, thought I should be strong enough to handle it, wanted to keep using, didn’t think the problem was serious enough, stopped on my own*
   - Were you ever uncomfortable about seeking help for your ice use because of what you thought others might think? (If yes, In what way/s?)
4. What other forms of support have you had? Family/friends?
   - How did that influence your treatment? Did it help you or stop you from engaging with treatment? In what ways?
   - How did those people support you?

**Feedback on program**

1. What were your expectations of the Ready2Change program prior to starting your support calls?
   - *Thought it would be a good idea to chat to someone*
   - *Not too intense, just on the phone*
2. What did you think of the Ready2Change Program?
   - *Counsellor*
   - *Content of sessions*
   - *Number of sessions*
   - *Workbooks*
3. What did you think of the telephone format?
   - *Convenience and flexibility*
   - *COVID social distancing*
4. How do you think it compares to face-to-face treatment?
   - Do you think you were more or less involved because it was over the phone? Do you feel like you were more accountable?
   - Did you have any problems with your phone? Like getting it charged or having enough credit or answering in time?
   - Where did you complete your calls? At home, work, in a park etc.
   - What were you doing during your calls?  Household tasks, working, smoking a cigarette etc. Did you find that helpful or distracting?
   - How did you prepare for calls? Did you have to do anything special like find a quiet place to call, find someone to look after kids?
   - What do you think about video conferencing?
5. If they had previous treatment*:* How did the Ready2Change program compare to any treatment you had before?

**Effectiveness of program**

1. What were you hoping to get out of the study when you started, in terms of your meth use?
   - *Reduce use*
   - *Stop completely*
   - *Why did you want that?*
2. How confident were you about making those changes?
3. What changes have you noticed since the Ready2Change program?
   - *Meth use*
   - *Other AOD use*
   - *Mental Health*
   - *Physical Health*
   - *Employment*
   - *Social connection (with family, friends, work, hobbies)*
4. Have you been able to maintain these changes [i.e. to meth use and/or other areas of life described by the participant in response to q12 and q14] since finishing the program?
   - If maintained: What have you been doing to maintain these changes?
   - If not maintained: Why do you think the changes did not continue after the program ended? What could have helped you to maintain these gains (e.g. more sessions, follow-up session, follow-up email or text, additional resources)
5. In what ways did the program help you?
6. In what ways did the program *not* help?
7. What aspects of the program helped you to achieve these changes?
   - *Counsellor*
   - *Telephone format*
   - *Booklets*
   - *Keeping track of your use*
   - *Identifying triggers and consequences*
   - *Managing urges (SOBER breathing)*
   - *Establishing a helpful routine*
8. What other things were going on for you that impacted how you took part in the program?
   - *Family (partner, parents, kids)*
   - *Friends*
   - *Employment*
   - *Housing*
   - *Forensic (court, drivers licence)*
9. Have you sought out any other treatment since participating in this program?
   - If so, what type?
   - If not, have you considered seeking out any other treatment since this program?
   - How has participating in Ready2Change influenced your likelihood of getting help for your substance use in the future?
10. How can we improve the R2C program to better support others with meth use problems?

**Implementation of program**

1. You had __ phone calls with *_________*, is that right? Why did you end up participating in fewer than the 6 calls typically offered by the program?
   - If had six calls: “What helped you participate in the whole program?
2. How much effort was involved in participating in the support calls?
   - Was it hard to do the support calls?
   - *Time, energy, calls going over or starting late, etc.)*
   - What kind of things did you have to give up?
3. What do you think about the way the support program was run?
   - *Structure of the program, content etc*
   - *Scheduling/rescheduling appointments*
   - *Counsellor contact attempts*
4. How do you think we should let people know about the program?
   - *Putting an ad on Facebook / other social media*
   - *Posters in public areas*
   - *Website*

**Conclusion**

1. Do you have any final thoughts you would like to share? Is there anything that I’ve missed that you would like to discuss?
