# Supplementary material for: Client Experiences of a Telephone‐Delivered Intervention for Methamphetamine Use Disorder
Source: Drug Alcohol Rev. 2026 Feb 6;45(2):e70108. doi: 10.1111/dar.70108 (PMC12880202; doi:10.1111/dar.70108)
Supplement: Supplementary file 3 — Supporting Information: S3: Researcher description. [file DAR-45-0-s003.docx]

**Supplementary Material 3:**

**Researcher description**

Several researcher characteristics are important to note. Interviewers were all women, employed as research assistants and had varied qualifications and backgrounds. All interviewers had Bachelor degrees in a related field (i.e. Psychology, Science). Interviewers worked on study recruitment, screening, and baseline data collection for the R2C-M trial and therefore eight participants had contact with their interviewer prior to the interview. Interviewers scheduled participants’ Ready2Change appointments and liaised with Ready2Change counsellors throughout the trial.
